# Sleep apnea in REM sleep as an amplifier of vascular contributions to Alzheimer's disease

**DOI:** 10.1002/alz.71735

**Published:** 2026-08-11

**Authors:** Destiny E. Berisha, Ruth M. Benca, Bryce A. Mander

**Affiliations:** ^1^ Department of Neurobiology and Behavior University of California, Irvine Irvine California USA; ^2^ Center for the Neurobiology of Learning and Memory University of California, Irvine Irvine California USA; ^3^ Department of Psychiatry and Behavioral Medicine Wake Forest University Winston‐Salem North Carolina USA; ^4^ Department of Cognitive Sciences University of California, Irvine Irvine California USA; ^5^ Department of Psychiatry and Human Behavior University of California, Irvine Irvine California USA; ^6^ Institute for Memory Impairments and Neurological Disorders University of California, Irvine Irvine California USA; ^7^ Department of Pathology and Laboratory Medicine University of California, Irvine Irvine California USA

**Keywords:** Alzheimer's disease, cerebrovascular disease, cognitive decline, dementia, hypoxemia, medial temporal lobe, obstructive sleep apnea, rapid eye movement sleep, sleep‐disordered breathing, white matter hyperintensities

## Abstract

Obstructive sleep apnea (OSA), a common though underdiagnosed disorder in older adults, represents a potential upstream vascular stressor linked to increased Alzheimer's disease (AD) risk. In this Perspective article, we propose a stage‐informed framework in which rapid eye movement (REM) sleep‐related respiratory events amplify vascular contributions to AD. This framework is unified across four linked lines of evidence suggesting that (1) REM sleep physiology magnifies the hypoxic and hemodynamic burden of OSA, (2) the cardiometabolic consequences of sleep apnea are heightened in REM sleep, (3) REM‐related hypoxemia is associated with markers of cerebrovascular pathology, and (4) vascular effects associated with REM‐related hypoxemia are associated with medial temporal lobe vulnerability and memory dysfunction: core features of AD. Although direct evidence linking REM‐specific OSA to AD biomarkers is lacking, we highlight REM sleep as a potentially critical window through which sleep‐disordered breathing may amplify vascular contributions to AD.

## Highlights

- Rapid eye movement (REM) sleep physiology amplifies hypoxic and arousal burden in obstructive sleep apnea (OSA).
- REM‐predominant OSA confers an elevated cardiometabolic risk.
- Markers of cerebrovascular pathology are linked to REM‐related hypoxemia.
- Vascular pathways link REM‐related hypoxemia to medial temporal lobe vulnerability.
- REM‐predominant OSA may represent a modifiable risk factor for Alzheimer's disease.

## 1 INTRODUCTION

Alzheimer's disease (AD) is the most common cause of dementia^1^ and, similar to cardiovascular disease, has a decades‐long preclinical incubation period with aging as a central risk factor.^2^ A growing body of evidence suggests that AD is a multifactorial disease in which vascular dysfunction may play an early and mechanistically meaningful role.^3^, ^4^ Obstructive sleep apnea (OSA), a prevalent yet underdiagnosed^5^ sleep disorder in older adults,^6^ is characterized by recurrent partial or total upper‐airway collapse during sleep, resulting in ischemia or hypoxia and hypoperfusion along with arousals, sympathetic activation, sleep fragmentation, and blood oxygen desaturation.^7^ OSA increases AD risk,^8^ with established evidence identifying OSA as an upstream vascular stressor with links to cardiometabolic risk factors and accelerators of AD neuropathology.^9^

Two complementary frameworks underscore vascular contributions to AD. First, the vascular contributions to cognitive impairment and dementia (VCID) framework emphasizes that vascular brain injury frequently co‐occurs with neurodegeneration and contributes to cognitive decline.^10^ In parallel, the vascular hypothesis of AD (VHAD) proposes that vascular dysfunction may precede and amplify classical AD pathology via mechanisms such as neurovascular uncoupling, blood–brain barrier (BBB) breakdown, chronic cerebral hypoperfusion, and impaired clearance of amyloid beta (Aβ).^10^ An influential mechanistic bridge between vascular dysfunction and AD is the two‐hit vascular hypothesis, wherein microvascular injury (“hit 1”) may promote amyloid accumulation through impaired clearance and BBB dysfunction, while amyloid deposition (“hit 2”) then exerts vasculotoxic effects that further damage the microvasculature, creating a vicious cycle.^11^ Although none of these frameworks alone explain AD pathogenesis, vascular dysfunction is increasingly recognized as a key modifier interacting with amyloid, tau, neuroinflammation, and genetic susceptibility to shape disease onset and progression.^10^

In 2012, Zimmerman and Aloia brought forth a framework by which sleep‐disordered breathing is linked to cognitive impairment via a microvascular model in which chronic intermittent hypoxemia leads to vasculopathy that is ultimately expressed as cognitive impairment in older adults.^12^ In this Perspective article, we extend this theory by proposing a stage‐informed framework in which rapid eye movement (REM) sleep‐related OSA functions as an amplifier of vascular contributions to AD. We synthesize evidence supporting four linked hypotheses: (1) REM sleep physiology magnifies the hypoxic and hemodynamic burden of OSA; (2) the cardiometabolic consequences of sleep apnea are heightened in REM sleep; (3) REM‐related hypoxemia (measured by various metrics such as REM minimum oxygen saturation (SpO_2_), percentage of REM sleep time with SpO_2_ < 90%, REM‐specific oxygen desaturation index, and REM hypoxic burden) is associated with markers of cerebral small vessel disease (CSVD), including white matter hyperintensities (WMHs); and (4) vascular effects associated with REM‐related hypoxemia may contribute to medial temporal lobe (MTL) vulnerability and memory dysfunction, which are early known consequences of AD pathogenesis.^13^ Although current evidence is insufficient to conclude that these effects are greater than those associated with OSA events occurring during non‐REM (NREM) sleep, existing findings support a mechanistic framework linking REM‐related respiratory events to the amplification of microvascular injury processes central to theories describing vascular contributions to AD.

This framework is likely one of several interacting mechanisms through which REM‐related OSA may influence AD risk. The current Perspective deliberately focuses on vascular contributions given that these remain comparatively underexplored, but future mechanistic work should aim to disentangle the relative and combined contributions of both vascular and non‐vascular pathways. Notably, similarly to AD risk, the burden and consequences of REM‐related respiratory events are not uniformly distributed across populations, with emerging evidence suggesting meaningful disparities by sex and race/ethnicity in these pathways that will be further discussed in the context of the highlighted studies.^14^, ^15^, ^16^, ^17^

## 2 Physiological and Cardiometabolic Consequences of Sleep Apnea in REM Sleep

**FIGURE 1:**
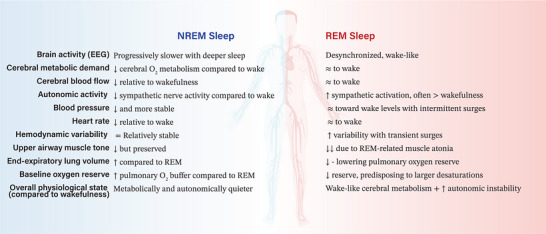
Physiological features of REM sleep that may amplify the vascular consequences of obstructive sleep apnea. Relative to NREM sleep, REM sleep is characterized by greater sympathetic activation, increased blood pressure variability, and more unstable ventilatory control. The combination of greater autonomic instability, impaired oxygen buffering, and higher neurometabolic demand during REM sleep may increase vulnerability to hypoxic and hemodynamic stress during obstructive respiratory events.^18^, ^19^, ^20^, ^21^, ^22^, ^23^, ^24^, ^25^ EEG, electroencephalography; NREM, non–rapid eye movement; O_2_, oxygen; REM, rapid eye movement.

Relative to NREM sleep, differences in ventilatory drive, lung O_2_ reserve, and neurometabolic demand create a distinct physiological environment during REM sleep that may magnify the downstream consequences of OSA (Figure 1). For example, REM sleep is associated with greater sympathetic activation and increased hemodynamic surges compared to NREM sleep.^18^ Direct microneurographic recordings further show that, whereas sympathetic nerve activity, blood pressure, and heart rate progressively decline across deeper stages of NREM sleep, REM sleep is characterized by a marked rebound in sympathetic activity to levels exceeding wakefulness, together with increased blood pressure variability and intermittent surges.^18^ It is also associated with distinct physiological patterns of upper airway function compared to NREM sleep wherein ventilation is more erratic and SpO_2_ less well maintained compared to NREM sleep,^19^ supported by evidence of transient bursts in which breathing rate can increase more than 2‐fold during REM sleep.^20^ Other studies have found greater reduction in ventilatory response to nocturnal hypoxemia during REM compared to that seen in NREM sleep.^21^ Reduced end‐expiratory lung volume during REM sleep^22^ decreases the pulmonary oxygen reserve, lowering baseline SpO_2_ and producing larger desaturation events during apneas than occur during NREM sleep.^23^ Higher metabolic demand in the brain during REM relative to NREM sleep may also increase vulnerability to oxygen desaturation during respiratory events.^24^ Consistent with this, human physiologic studies demonstrate that deep NREM sleep is accompanied by substantial reductions in cerebral blood flow (CBF) and cerebral oxygen metabolism, whereas REM sleep returns to near wake‐like levels of CBF and cerebral metabolic demand.^25^ Thus, compared to NREM sleep, REM sleep appears to combine greater autonomic instability with relatively higher cerebral energetic demand, a physiological context that may heighten susceptibility to the hypoxic and hemodynamic consequences of obstructive respiratory events. The sustained mismatch between oxygen supply and metabolic demand promotes oxidative stress, neuroinflammation, and BBB degradation, all of which can facilitate the accumulation of neurotoxic substrates and accelerate neurodegenerative cascades that are central to the VHAD model.^3^

OSA severity is most often indexed by the apnea–hypopnea index (AHI), which is calculated by dividing the total number of apneas (breathing cessation events) and hypopneas (shallow breathing events) by the total hours of actual sleep. A key limitation of the AHI is that it collapses respiratory events across all sleep stages into a single summary metric (see Text Box 1), though stage‐specific indices can be calculated. REM AHI and NREM AHI are derived by dividing the number of respiratory events occurring within each respective stage by the total time spent in that stage. Accordingly, studies using stage‐specific measures increasingly show that obstructive events in REM versus NREM sleep carry distinct associations with clinical outcomes. For example, a longitudinal community‐based cohort of 1451 middle‐aged adults from the Wisconsin Sleep Cohort showed that REM AHI, but not NREM AHI, was independently associated with both prevalent (odds ratio [OR] for REM AHI ≥ 15 vs. < 1: 1.26, 95% confidence interval [CI]: 1.01–1.57) and incident hypertension (OR: 1.77, 95% CI: 1.08–2.92) after adjustment for body mass index (BMI) and major cardiovascular risk factors.^26^ A dose–response relationship with prevalent hypertension was also evident among individuals without NREM OSA (NREM AHI ≤ 5), particularly when hypertension was defined by ambulatory blood pressure monitoring.^26^ In longitudinal analyses restricted to this subgroup, REM AHI ≥ 15 was associated with nearly 2‐fold greater odds of developing hypertension over time relative to REM AHI < 1 (OR: 1.98, 95% CI: 1.01–3.88).^26^

**Text Box 1: Limitations of AHI and the Case for Stage‐Specific Hypoxic Burden Metrics**

**Table:** 

Traditionally, obstructive sleep apnea (OSA) severity is indexed by the apnea–hypopnea index (AHI), which reflects the average frequency of breathing events per hour across the total sleep period. Because non–rapid eye movement (NREM) sleep constitutes the majority of total sleep time, summary metrics such as the overall AHI or respiratory disturbance index (RDI) use total sleep time as their denominator, which dilutes the contribution of events concentrated in rapid eye movement (REM) sleep, given that REM comprises only roughly 20% to 25% of the night. As a result, the AHI may systematically underestimate physiologically meaningful exposure when respiratory events occur predominantly during REM sleep. Crucially, the AHI does not capture the severity, duration, or cumulative dose of oxygen desaturation associated with respiratory events, nor the duration and severity of associated arousals and their attendant sympathetic surges. These dimensions are particularly relevant because apneas and hypopneas during REM sleep tend to be longer and associated with greater oxygen desaturation than NREM events,^19^ meaning event frequency alone inadequately reflects the severity of the respiratory disturbance. Compounding these measurement issues, the lack of a standardized definition of REM‐predominant OSA, with varying AHI thresholds, REM‐sleep duration requirements, and ratio‐based criteria, presents a major challenge to comparability across studies (see Text Box 2).^31^ Stage‐specific hypoxia metrics offer several advantages: **REM minimum oxygen saturation (SpO_2_)**: captures the nadir of oxygen saturation during REM sleep, reflecting peak hypoxic stress. **Percentage of REM sleep time with SpO_2_ < 90%**: indexes cumulative hypoxic dose specifically during REM, independent of event frequency. **REM‐specific oxygen desaturation index (REM ODI)**: counts clinically meaningful desaturation events (typically 3% or 4% drops) per hour of REM sleep. **REM hypoxic burden**: integrates the depth and duration of desaturations during REM sleep into a single continuous metric (using an area‐under‐the‐curve approach), capturing physiological exposure beyond simple event counts.

**Text Box 2. Operational Definitions of Rapid Eye Movement–Predominant Obstructive Sleep Apnea**

**Table:** 

No consensus definition of rapid eye movement (REM)‐predominant obstructive sleep apnea (OSA) currently exists, and variability across studies limits comparability of prevalence estimates and outcome associations. The choice of definition meaningfully affects prevalence estimates. As outlined in a comprehensive discussion on this topic by Varga and Mokhlesi, applying only the REM/non–rapid eye movement (NREM) ratio criterion identified REM‐predominant OSA in 36.7% of a clinical sample, whereas adding a NREM apnea–hypopnea index (AHI) ceiling of eight events/hour reduced prevalence to 13.5% in the same cohort.^31^ Women consistently show higher prevalence of REM‐predominant OSA than men across definitions, underscoring the importance of definitional standardization for sex‐stratified research.^31^ Criteria often include some combination of the following: A ratio of REM AHI to NREM AHI of ≥ 2, often accompanied by a minimum REM sleep duration requirement (typically 30 minutes) and a minimum overall AHI threshold (usually 5 events/hour). An absolute REM AHI threshold (e.g., 15 or 30 events/hour) regardless of NREM AHI. A combined criterion requiring both an elevated REM AHI/NREM AHI ratio and a NREM AHI ceiling (e.g., NREM AHI < 8 or 15 events/hour) to exclude cases in which substantial NREM OSA co‐occurs.

Similarly, in a community‐based sample of 739 middle‐aged and older men without previously diagnosed OSA, men with severe REM OSA (REM AHI ≥ 30/hour) had roughly 2.4‐fold higher odds of prevalent hypertension (OR: 2.40, 95% CI: 1.42–4.06) and 2.2‐fold higher odds of recent‐onset hypertension (OR: 2.24, 95% CI: 1.04–4.81) than men with REM AHI < 10/hour, independent of NREM AHI, waist circumference, and other cardiovascular risk factors.^27^ Notably, REM OSA remained associated with prevalent hypertension (at REM AHI ≥ 20/hour) even among men with an overall AHI < 10.^27^ Other studies have also shown that greater OSA severity during REM sleep showed a dose–response association with higher risk of incident systolic and diastolic nocturnal non‐dipping blood pressure, independent of NREM AHI.^28^ This also extends to morning blood pressure, as higher REM AHI, but not NREM AHI, has been independently associated with morning hypertensive blood pressure levels after adjustment for age and BMI^29^ in adults referred for suspected OSA, further supporting a potential link unique to REM sleep between obstructive respiratory events and the vascular stressors described in the two‐hit hypothesis. Given that hypertension has been linked to greater AD neuropathological change at autopsy, specifically higher plaque and tangle burden,^30^ REM OSA‐associated hypertension may represent one plausible route through which respiratory events in REM sleep might amplify vascular contributions to AD. The cardiovascular consequences of OSA severity in REM sleep have been summarized in a comprehensive review by Varga and Mokhlesi^31^ and in another by Alzoubaidi and Mokhlesi.^32^

Stage‐dependent findings extend beyond cardiovascular relationships to broader cardiometabolic associations. In a cohort of 115 adults with type 2 diabetes, REM AHI, but not NREM AHI, was independently associated with worse long‐term glycemic control, showing a dose–response increase in adjusted glycated hemoglobin (HbA1c) from 6.3% (lowest REM AHI quartile) to 7.3% (highest quartile).^33^ Similarly, in a large community‐based cross‐sectional study of middle‐aged and older adults from the Sleep Heart Health Study, REM AHI was independently associated with greater insulin resistance (*β* = 0.04, 95% CI: 0.01–0.07), whereas NREM AHI was associated with fasting glucose levels (*β* = 0.93, 95% CI: 0.14–1.72) and post‐challenge glucose levels (*β* = 3.0, 95% CI: 0.5–5.5) after adjustment for adiposity and sleep duration, among other factors.^34^ In a small exploratory longitudinal study of women with REM‐predominant OSA followed over 60 months, individuals with REM‐predominant OSA (defined as AHI ≥ 5 with an AHI_REM_/AHI_NREM_ ratio ≥ 2; see Text Box 2 for operational definitions of REM‐predominant OSA) showed worsening arterial stiffness and deterioration in glucose metabolism despite continuous positive airway pressure (CPAP) or oral appliance therapy.^35^ Disruption of glucose metabolism is particularly relevant to AD, as cerebral glucose hypometabolism is an early and prominent feature of AD and amnestic mild cognitive impairment (MCI).^36^, ^37^ Glucose delivery to the brain depends on vascular transport and insulin‐mediated cellular use, processes that are impaired in systemic insulin resistance, which is also an established risk factor for AD.^38^ Together, these findings raise the possibility that REM‐related OSA may contribute to AD vulnerability not only through peripheral systemic vascular factors, but also through metabolic pathways that promote insulin resistance and impaired neuronal energy use.

## 3 CSVD and OSA in REM Sleep

Cerebrovascular pathology, depending on the type, can act independently or synergistically with AD, contributing to neurodegeneration.^39^, ^40^ WMHs, a common marker of CSVD linked to vascular dysfunction and dementia risk, are strongly associated with cognitive impairment in aging and AD.^41^ Beyond overt cardiovascular disease, peripheral vascular factors such as arterial stiffness may contribute to WMH formation by increasing CBF pulsatility and impairing cerebrovascular regulation, two mechanisms central to the development of CSVD.^41^

Intermittent hypoxia appears to play a critical role in the relationship between OSA and CSVD. Chronic mild hypoxia triggers transient BBB disruption in healthy rodent models and initiates angiogenic remodeling and microglial activation and aggregation around leaky vessels.^42^ Notably, these rodent studies demonstrate an age‐related effect, as BBB disruption under hypoxic conditions was markedly greater in aged mice than in young mice, likely due to delayed vascular remodeling and reduced microglial vasculo‐protective responses.^43^ Consistent with these rodent findings, human studies have reported associations between nocturnal hypoxemia and CSVD in aging populations. In a longitudinal community‐based cohort study of 2150 middle‐aged to older Hispanic/Latino adults with sleep assessments preceding neuroimaging by ≈ 10 years, lower mean nocturnal oxygen saturation (mean SpO_2_) during sleep was associated with greater WMH volumes on subsequent magnetic resonance imaging (MRI; β = –0.095, 95% CI: −0.164 to −0.025) after adjustment for demographic and vascular risk factors.^44^ Similarly, in a prospective community‐based cohort study of 587 middle‐aged to older adults from the Multi‐Ethnic Study of Atherosclerosis, the authors calculated hypoxic burden as the total area under the respiratory event‐related oxygen desaturation curve divided by total sleep time.^45^ They found that 1 standard deviation (SD) increase in hypoxic burden was associated with a 0.09 SD increase in WMH volume after adjustment for demographic characteristics, cardiovascular risk factors, and comorbidities (95% CI: 0.01–0.17).^45^ This was in contrast to measures of ventilatory burden, arousal burden, and conventional OSA metrics which were not significantly associated with WMH volume, further suggesting that nocturnal hypoxemia, rather than sleep fragmentation or respiratory event frequency, may be the specific OSA‐related correlate of WMH burden.^45^

Beyond feature specificity, emerging evidence has increasingly drawn sleep stage‐specificity connections as well, linking REM‐related respiratory events to increased WMH burden. In a cross‐sectional study of 842 ethno‐racially diverse community‐dwelling older adults from the Health and Aging Brain Study: Health Disparities (HABS‐HD) cohort, higher REM AHI remained independently associated with greater WMH volume (OR per 1 SD increase = 1.19, 95% CI: 1.03–1.38) even after adjustment for cardiovascular risk factors and demographic characteristics.^46^ Notably, no robust associations were observed for overall AHI nor NREM AHI, supporting a specific role of REM sleep in the relationship between sleep‐disordered breathing and CSVD.^46^ As adjustment for hypertension did not materially change the REM AHI–WMH association,^46^ it is plausible that REM‐specific breathing events may play a role in WMH development beyond effects on cardiovascular risk alone. In our observational study of 37 cognitively unimpaired older adults, we similarly showed that lower minimum sleep SpO_2_ and greater time spent with SpO_2_ < 90% significantly predicted greater global WMH burden, whereas global OSA frequency metrics such as AHI and oxygen desaturation index (ODI) did not.^47^ Critically, when hypoxemia was parsed by sleep stage, the association with WMH was driven by REM sleep hypoxemia (i.e., REM sleep minimum SpO_2_ remained a significant predictor of global WMH volume whereas NREM minimum SpO_2_ did not^47^). These relationships also showed regional specificity, by which REM minimum SpO_2_ predicted frontal and parietal but not temporal or occipital WMH burden.^47^ Collectively, these findings provide additional support for the hypothesis that REM‐related respiratory disturbances may play a role in processes implicated in the development of CSVD.

Other markers of cerebrovascular pathology appear to be associated with REM‐related events in OSA as well. In a retrospective study of 318 adults with ischemic cerebral infarction and OSA (AHI ≥ 5), patients were classified as having REM‐predominant OSA (AHI_REM_/AHI_NREM_ ≥ 2 with at least 30 minutes of REM sleep) or NREM‐predominant OSA (AHI_REM_/AHI_NREM_ < 2). Those with REM‐predominant OSA showed poorer functional recovery at 3 months, demonstrated by higher modified Rankin Scale scores, greater systemic inflammation, and a significantly higher prevalence of basal ganglia infarction.^48^ Notably, baseline demographic and vascular risk factors were broadly similar between groups. Importantly, as expected,^19^ individuals with REM‐predominant OSA demonstrated more severe REM‐specific hypoxemia, including lower nadir oxygen saturation and longer apnea duration, despite having a lower overall AHI.^48^ As the basal ganglia are supplied by small penetrating end arteries (i.e., lenticulostriate branches of the middle cerebral artery) that lack sufficient collateral circulation, it is notably highly vulnerable to hypoxia, blood pressure fluctuations, arterial stiffness, and endothelial dysfunction.^49^ Taken together, these findings further support the notion that respiratory disturbances in REM sleep may contribute to region‐specific cerebrovascular injury independent of total OSA severity and cardiovascular risk factors.

A comprehensive review has previously synthesized the literature on the cerebrovascular consequences of OSA, underscoring that OSA is not only linked to cardiovascular disease broadly, but also exerts specific, clinically important effects on the cerebral circulation and cerebrovascular outcomes.^50^ Durgan and Bryan summarized epidemiologic data showing OSA is an independent risk factor for stroke and is highly prevalent in stroke patients.^50^ Mechanistically, they describe how apneic events produce large, rapid swings in blood pressure and blood gases that drive marked fluctuations in CBF velocity,^50^ particularly during longer apneas.^51^ Typically, obstructive apneas and hypopneas in REM sleep are longer in duration and are associated with significantly greater oxygen desaturation compared to events occurring during NREM sleep,^19^ which is likely explained by a higher tendency for upper airway collapse compared to NREM sleep due to cholinergic‐mediated suppression of genioglossus activity.^52^ As such, chronic, untreated respiratory events in REM sleep may contribute to gradual loss of adaptive physiology underlying cerebrovascular architecture to cope with ischemic events over time. Chronic OSA is associated with lower resting CBF, impaired cerebrovascular autoregulation, and reduced vascular reserve: all changes that can leave the brain more vulnerable to ischemia, thereby creating a feed‐forward loop.^50^ Even during wakeful rest, individuals with OSA exhibit reduced regional CBF, demonstrated using both arterial spin labeling MRI^53^ and single photon emission computed tomography (SPECT) imaging.^54^ Extending this, Baril et al. showed that a greater burden of respiratory events during REM sleep was linked to reduced daytime resting CBF in ventromedial prefrontal and fronto‐insular regions, whereas events during NREM sleep were associated with hypoperfusion in left sensorimotor and temporal cortices, suggesting sleep stage–specific patterns of CBF deficits in OSA.^55^ Notably, REM‐related events were still linked to fronto‐insular hypoperfusion even among participants with overall mild OSA (AHI < 15).^55^

Reductions in baseline CBF are widely recognized as early changes that appear in individuals with AD,^4^ wherein greater CBF deficits appear to track strongly with worse cognitive outcomes.^56^ Converging evidence also suggests that chronic cerebral hypoperfusion is associated with WMH burden and may represent an important vascular pathway in AD.^57^ Additionally, imaging and experimental data indicate that regional hypoperfusion, impaired cerebrovascular reactivity, and BBB dysfunction precede overt amyloid and tau pathology and may represent some of the earliest detectable events in the AD cascade.^3^ Within the framework of the two‐hit vascular hypothesis, sustained reductions in CBF constitute an Aβ‐independent “hit” that compromises neurovascular unit integrity, disrupts endothelial‐dependent vasodilation, promotes oxidative stress, and impairs pericyte‐mediated capillary regulation.^3^ These vascular insults can initiate a feed‐forward process by reducing clearance of Aβ across the BBB, increasing local amyloid production under hypoxic conditions, and exacerbating tau phosphorylation through ischemia‐related signaling pathways.^3^ Over time, Aβ accumulation then exerts its own vasculotoxic effects by inducing endothelial dysfunction, vascular smooth muscle hypercontractility, and further reductions in cerebrovascular reactivity thereby amplifying hypoperfusion and neurovascular uncoupling.^11^ In this context, repeated REM‐related hypoxemia and hemodynamic instability in OSA may act as a chronic vascular stressor that can contribute to processes implicated in both Aβ‐independent and Aβ‐dependent neurodegenerative pathways. By promoting endothelial injury, pericyte dysfunction, oxidative stress, and impaired autoregulatory capacity, REM‐related OSA events may lower the brain's resilience to ischemic challenge and potentiate the vascular mechanisms increasingly recognized as central to early AD pathogenesis and cerebrovascular pathology. Collectively, findings across community‐based samples and clinical cohorts converge on a consistent pattern in which REM‐related respiratory events and hypoxemia measures, including REM minimum SpO_2_, REM ODI, and REM‐specific desaturation burden, are associated with greater WMH burden and other markers of cerebrovascular pathology. Notably, these associations persist after adjustment for traditional cardiovascular and demographic risk factors and often remain when NREM sleep and global OSA severity metrics (e.g., overall AHI) are not significant.

## 4 MTL Vulnerability and Memory

Regions of the MTL, the seat of learning and memory in the brain, show some of the earliest histopathological changes in AD.^58^ Recent evidence underscores that MTL atrophy is also strongly influenced by cerebrovascular pathology, particularly by WMHs, suggesting that the early vulnerability of the MTL likely reflects a convergence of aging and disease processes.^59^ Whereas classical AD pathologies, particularly neurofibrillary tangles, contribute to MTL degeneration, vascular brain injury indexed by WMHs may meaningfully amplify or partially explain age‐related MTL atrophy, consistent with a vascular‐mediated pathway to MTL neurodegeneration.^39^ In support of this, a study examining a large cohort of both cognitively unimpaired and cognitively impaired individuals showed that MTL atrophy remained associated with age even after accounting for amyloid, tau, and alpha‐synuclein pathology, while WMHs emerged as the strongest and most consistent predictor of MTL atrophy and mediated a substantial portion of the age–MTL atrophy association.^39^ More broadly, these findings suggest that cerebrovascular pathology may contribute to neurodegeneration either independently of, or synergistically with, classical AD pathology. In this framework, vascular injury affecting MTL networks may reduce the structural resilience of the MTL, thereby amplifying the impact of AD‐related processes and potentially accelerating the emergence of cognitive symptoms.

The MTL appears to be particularly vulnerable in individuals with OSA.^60^ Neuroimaging studies have shown that individuals with OSA exhibit reduced global CBF and regional hypoperfusion in temporal cortical areas and the hippocampus, with the severity of oxygen desaturation being associated with reduced perfusion in temporal regions.^61^ Complementary to findings in humans, work in rodent models has shown that hypertension‐mediated hypoxia, a relevant model for OSA, is associated with BBB leakage and neurodegeneration in cortical and hippocampal brain regions, including increased tau pathology in cornu ammonis 3 (CA3) and the dentate gyrus regions of the hippocampus.^62^

Recent evidence suggests that hypoxemia during REM sleep may be associated with greater MTL structural vulnerability and impaired MTL function, and that cerebrovascular pathology might mediate these relationships.^47^, ^63^ Consistent with this framework, in our observational study of 37 cognitively unimpaired older adults, we found that worse hypoxemia (as indexed by minimum sleep SpO_2_) in REM sleep, but not NREM sleep, predicted greater WMH volumes, which in turn were associated with decreased hippocampal volumes and entorhinal cortical thickness.^47^ Greater frontal WMH burden indirectly mediated the relationship between REM hypoxemia and entorhinal thickness in this cohort.^47^ Importantly, thinner entorhinal cortical thickness was in turn associated with poorer overnight retention of mnemonic discrimination performance, a measure of hippocampal‐dependent memory.^47^

Similarly, in our cross‐sectional study of 81 cognitively unimpaired middle‐aged and older adults enriched for AD risk^64^ (≈ 32% apolipoprotein E [*APOE*] ε4 carriers; ≈ 69% with a parental history of AD), participants were given the Rey Auditory Verbal Learning Test (RAVLT), a hippocampal‐dependent test of verbal episodic memory.^65^ We found that REM ODI remained associated with worse total learning (β = −7.91, *p* = 0.02) and worse long‐delay recall (β = −2.46, *p* = 0.02).^64^ The corresponding NREM indices were not significant predictors of either outcome.^64^ For delayed recall, the REM ODI association was significantly stronger than its NREM counterpart, supporting sleep‐stage specificity rather than a global OSA effect.^64^ Moderation analyses further indicated that the association between REM ODI and total learning was most pronounced in adults aged ≥ 60 (β = −0.47, *p* < 0.05), whereas *APOE* ε4 carriers specifically drove the associations between the REM:NREM ODI ratio and total learning (β = −18.17, *p* < 0.01), suggesting that hypoxemia during REM sleep may be especially relevant for memory in groups at higher risk for AD.^64^

Complementing these findings, Lam et al. examined 338 middle‐aged and older adults with subjective cognitive complaints or MCI recruited from a memory‐clinic setting, a subset of whom completed high‐resolution hippocampal MRI alongside overnight polysomnography (PSG).^63^ In the full cohort, REM hypoxemia was indexed as REM ODI (number of ≥ 3% desaturations per hour of REM sleep), and verbal learning and delayed recall were assessed with the RAVLT.^63^ Although REM ODI was not directly correlated with either verbal learning or delayed recall at the whole‐sample level, higher REM ODI was associated with smaller hippocampal cornu ammonis 1 (CA1) subfield volume, which was in turn associated with poorer verbal learning and delayed recall.^63^ Mediation analyses confirmed a significant indirect effect of REM ODI on both verbal learning (β = −0.09, 95% CI: −0.238 to −0.005) and delayed recall (β = –0.10, 95% CI: –0.255 to –0.005) through CA1 volume.^63^ Critically, REM specificity was explicitly tested by repeating the analysis for NREM ODI, which showed no significant associations with hippocampal subfields, consistent with a REM‐specific pathway, though the authors did not directly test whether the indirect effect was moderated by sleep stage.^63^ Taken together, these studies point to a consistent link between REM sleep hypoxemia and both MTL vulnerability and impaired memory.

## 5 CONCLUSION

**FIGURE 2:**
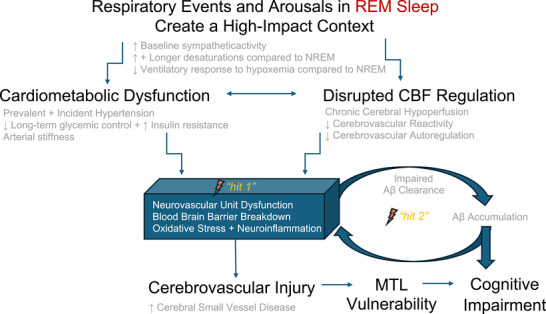
Proposed framework linking REM sleep‐related obstructive sleep apnea to vascular contributions to Alzheimer's disease. This schematic illustrates the conceptual model proposed in this Perspective within the framework of vascular contributions to cognitive impairment and dementia and the two‐hit vascular hypothesis of Alzheimer's disease. The distinct physiological environment of REM sleep including greater sympathetic activation, reduced ventilatory stability, diminished pulmonary oxygen reserve, and higher cerebral metabolic demand may magnify the hypoxic and hemodynamic burden of OSA. Recurrent REM‐related hypoxemia and blood pressure surges may promote vascular and metabolic dysfunction, including hypertension, insulin resistance, endothelial injury, and oxidative stress. These processes contribute to cerebrovascular pathology, which may increase vulnerability of medial temporal lobe networks, amplifying pathways implicated in Alzheimer's disease. Aβ, amyloid beta; CBF, cerebral blood flow; MTL, medial temporal lobe; NREM, non–rapid eye movement; OSA, obstructive sleep apnea; REM, rapid eye movement.

Collectively, the evidence synthesized in this Perspective supports the view that REM sleep–specific sleep‐disordered breathing represents an understudied yet biologically plausible factor in vascular contributions to AD risk. The evidence reviewed here supports a sequential framework in which the distinct physiological environment of REM sleep amplifies the cardiovascular and hypoxic burden of OSA, potentially creating conditions favorable to CSVD‐related processes across diverse populations. These vascular changes may in turn promote MTL structural degeneration and impair episodic memory ability, positioning REM‐related respiratory events as a potentially modifiable pathway linking sleep apnea to AD risk and cognitive decline more broadly (Figure 2).

Despite mounting evidence linking sleep‐disordered breathing to dementia risk,^8^ it remains unknown whether treatment of REM‐predominant sleep apnea alters the underlying CSVD trajectories thought to be associated with that risk. Moreover, the extent to which hypoxia during REM sleep contributes to downstream AD pathophysiology, either independently or synergistically with amyloid, tau, and neuroinflammation, remains largely unexplored. This is plausible given a longitudinal study of older adults found that decrements in REM proportion and greater REM latency were both associated with a higher risk of incident dementia after a mean follow‐up of 12 years, while no relationships were detected with NREM stages.^66^ The study authors attributed this finding to a possible decrease in cholinergic activity known to occur in the early stages of AD.^66^ However, exclusion of participants with frequent hypopnea‐related arousals substantially attenuated the estimated dementia risk associated with reduced REM sleep duration, further implicating OSA‐related mechanisms in the link between REM sleep disruption and dementia risk.^67^

These unanswered questions may be particularly salient for populations that exhibit heightened vulnerability to both AD and sleep‐disordered breathing, including women^17^, ^68^ and Black adults^69^ suggesting that REM‐related apneic event mechanisms may contribute to observed disparities in cognitive aging and dementia incidence. For example, women tend to have a higher proportion of REM‐related respiratory events and a greater prevalence of REM‐predominant OSA compared to men.^15^ Importantly, aging further exacerbates these differences: while women often have lower overall AHI values, the prevalence and severity of OSA increases substantially after menopause.^16^ Disparities in OSA burden and dementia risk further underscore the need for a stage‐specific framework. Black adults, particularly Black women, show higher REM OSA burden, even when overall all‐night OSA metrics are similar to non‐Hispanic White adults.^17^ Black adults experience disparities in OSA burden, including higher prevalence in some age groups and greater disease severity in clinical cohorts,^14^ as well as elevated vascular risk factor burden.^70^ Among individuals with dementia, Black individuals differ from non‐Hispanic White individuals in dementia risk factor profiles and symptomatic presentation, including greater cognitive impairment severity.^69^ Given evidence that amyloid burden alone may incompletely explain dementia risk in Black individuals,^71^ REM‐related hypoxemia and associated cardiometabolic dysfunction and vascular injury represent plausible contributors to these disparities and warrant focused investigation. Future studies should prioritize stratified analyses by sex and race/ethnicity and ensure adequate representation of women and minoritized groups to determine whether REM‐specific mechanisms contribute differentially to AD risk across these populations. While a study of 842 participants from diverse racial and ethnic backgrounds found no statistically significant differences by race or ethnicity in the associations of REM AHI with WMH volume, stratified analyses suggested that the associations of REM AHI and oxygen desaturation with WMH volume were numerically stronger among Hispanic participants, raising the possibility of greater vulnerability in this population.^46^ Accumulating evidence suggests that the consequences of respiratory disturbances in REM sleep are unlikely to be uniform across individuals but instead may be amplified in populations already vulnerable to AD‐related neurodegeneration.^64^ *APOE* ε4 carriers, amyloid‐positive individuals, and those with a parental history of AD may exhibit heightened metabolic and vascular susceptibility to hypoxemia, rendering memory‐related circuits particularly vulnerable to REM‐specific insults.^64^ As such, it is possible that respiratory disturbances in REM sleep may intersect with genetic, sex‐related, vascular, and social determinants of health to shape AD vulnerability across the lifespan, though this remains to be investigated in detail. By drawing a mechanistic throughline from REM sleep–specific respiratory events to CSVD and MTL dysfunction, we aimed to highlight REM sleep as a critical and underappreciated context in which sleep apnea may accelerate vascular contributions to AD.

Beyond the vascular route emphasized here, REM‐related OSA may also impair REM‐dependent memory consolidation^72^ and REM‐associated synaptic homeostasis processes.^73^ In addition, REM‐related arousals and associated sympathetic surges may contribute to cerebrovascular and neuronal injury through pathways at least partially independent of hypoxemia and are particularly deleterious in aging populations. Another route that warrants cautious consideration is sleep‐dependent glymphatic clearance of neurotoxic waste; although most current evidence links glymphatic function predominantly to NREM sleep,^74^ it remains possible that REM‐related OSA disrupts this process, and clarifying any REM‐specific role represents an important direction for future work. These parallel pathways are not mutually exclusive with the vascular framework presented here; rather, they may interact and amplify one another, particularly in individuals with elevated AD risk.

A distinct consideration, conceptually opposite to the pathway proposed here, is reverse causality: advancing AD pathology could itself alter breathing and sleep architecture.^75^ This possibility cannot be definitively excluded using cross‐sectional, observational data. However, available evidence suggests that the effects of AD pathology on respiratory control emerge largely in late‐ and end‐stage disease, once pathology reaches brainstem and medullary respiratory centers such as the pre‐Bötzinger complex.^75^ As the OSA–AD associations of interest here are observed during preclinical and early disease stages, it is unlikely that they are driven primarily by AD pathology degrading respiratory control. We therefore regard reverse causality as a possibility that cannot be fully excluded but that current biological evidence does not strongly support during the preclinical window most relevant to this framework.

The emerging evidence presented here highlights the importance of sleep stage–specific measures of hypoxic burden and arousals in characterization and discovery of mechanisms driving the effects of OSA on AD risk. This is critical, as repetitive arousals increase sympathetic predominance, and this effect appears to be associated with the amount of REM sleep.^76^ However, the extent to which REM‐related arousals independently contribute to cerebrovascular injury and cognitive decline remains unclear, as conventional OSA metrics such as AHI do not capture the burden or severity of electroencephalography (EEG)‐defined arousals. Clarifying the stage‐specific contributions of sleep fragmentation, particularly REM‐related arousals, represents an important direction for future work aimed at disentangling the relative roles of hypoxemia and arousal‐related sympathetic activation in OSA‐related brain injury. Mechanistic studies assessing EEG‐derived arousal burden stratified by sleep stage will be essential for isolating the independent contributions of REM‐specific arousals versus hypoxemia to vascular injury. Additionally, reliance on single‐night studies in research may also underestimate the frequency and severity of events occurring in REM sleep, as one study found a statistically significant increase in REM sleep on the second night of PSG,^77^ likely due to the “first night effect” observed when quantifying sleep with clinical equipment in laboratory environments. Interestingly, this effect may even extend beyond the first night for REM‐related measures specifically, which have been shown to stabilize more slowly than other sleep parameters, often requiring more than one adaptation night.^78^

Preliminary evidence from a small exploratory study suggests that sustained CPAP use in individuals with AD and OSA may attenuate cognitive decline and improve sleep outcomes,^79^ though experimental studies are required to determine whether reductions in REM‐related hypoxemia account for trajectories of cerebrovascular injury, neurodegeneration, and cognitive decline to a greater extent than concurrent reductions in NREM‐related hypoxemia and arousals. Addressing these gaps will require longitudinal, biomarker‐informed studies that compare REM‐ and NREM‐predominant OSA phenotypes, validate REM‐specific hypoxic burden metrics, and deliberately include populations at elevated risk for AD, including women, *APOE* ε4 carriers, amyloid‐positive individuals, and historically underrepresented racial and ethnic groups. Priority study designs should include longitudinal cohorts with repeated PSG incorporating stage‐specific hypoxemia metrics alongside AD biomarkers including biofluid markers of AD‐related proteins and neuroinflammation. Studies comparing the effect of improving REM versus NREM hypoxemia (for example, through CPAP adherence data focused on the latter part of the night) on CSVD progression and cognitive trajectories will also inform this framework further.

Importantly, the conclusions of this Perspective are tempered to vascular contributions to AD given that, despite converging evidence linking REM‐related hypoxemia to CSVD and MTL vulnerability, there are currently no studies demonstrating that REM‐specific respiratory event burden predicts incident AD or accelerates AD biomarker trajectories (e.g., amyloid, tau, or neurodegeneration markers) independent of overall OSA severity. Second, much of the REM‐focused literature is cross‐sectional and relies on single‐night PSG, limiting causal inference and potentially underestimating REM OSA due to night‐to‐night variability. Finally, heterogeneity in how REM‐predominant OSA is defined across studies complicates direct comparisons and meta‐analytic synthesis.

Ultimately, reframing REM sleep as a critical window of cerebrovascular vulnerability offers a promising opportunity to refine risk stratification, personalize OSA treatment, and potentially intervene on a modifiable contributor to AD during its long preclinical course. These considerations carry potential clinical implications. REM‐predominant OSA may merit evaluation in future work as a subgroup for tailored treatment thresholds in older adults and those at elevated AD risk, even when overall AHI falls in the mild range (i.e., AHI < 15). Additionally, routine PSG scoring and reporting in both clinical practice and research should incorporate REM‐specific indices, such as REM AHI and REM‐related oxygen desaturation metrics, to enable more precise phenotyping and risk stratification. The 2024 Lancet Commission on dementia prevention, intervention, and care indicated that nearly half of dementias worldwide could theoretically be prevented by eliminating 14 risk factors, roughly 7 of which are cardiovascular: hypertension, obesity, excessive alcohol use, smoking, diabetes, physical inactivity, and high low‐density lipoprotein cholesterol, the latter a new addition from the previous report.^80^ As REM‐predominant OSA appears to confer an elevated cardiovascular risk, it may represent an additional pathway through which sleep‐disordered breathing exacerbates dementia risk. Evidence that REM‐related hypoxemia is associated with CSVD even after accounting for cardiovascular risk factors^47^ raises the possibility of parallel, partially independent mechanisms linking REM sleep apnea to neurodegeneration that warrants further study.

## AUTHOR CONTRIBUTIONS

**Destiny E. Berisha**: Conceptualization; writing—original draft; **Bryce A. Mander**: Conceptualization; writing—review & editing; **Ruth M. Benca**: Writing—review & editing.

## CONFLICT OF INTEREST STATEMENT

D. E. Berisha reports no disclosures relevant to the manuscript. R. M. Benca has served as a consultant to Alkermes, Eisai, Haleon, and Seaport Therapeutics. B. A. Mander has served as a consultant for Eisai Co., Ltd and currently serves on a scientific advisory board for AstronauTx, Ltd. Author disclosures are available in the Supporting Information.

## Supporting information



Supporting Information
